# St. Dunstan's—And after

**Published:** 1919-11-29

**Authors:** 


### THE FUTURE OF THE BLIND.

### IV. St. Dunstan's—and After.

The duties of the writer of these articles had several times taken him to certain departments of that complex organisation known as St. Dunstan's Hostel for Blinded Soldiers and Sailors, but he did not see the whole of it at one visit until quite late in the day, before which comprehensive visit he had mentioned his lack of first-hand knowledge to one of the officials. This gentleman told him that he should see St. Dunstan's before the training stage of the work had terminated, as "there was never anything like it in the world before, and there never will be again." The enormous enthusiasm and pride of the least worker of St. Dunstan's is indicative of its spirit, and is an important factor in the secret of its success. Every one of the workers is imbued with this spirit, and thinks that his or her department is one of the most interesting and important, if not the most interesting and important.

Anyone who has not visited this unique centre of industry triumphing over blindness should do so soon. In the nature of things this stage of the work must terminate in a comparatively short space and lead to the longer phase of the history of the organisation, to which we shall refer presently. It has already been described in these columns how in one institution 700 to 800 blind men are eagerly undergoing training in massage, office work, including shorthand and typewriting, telephone operating, basket-making, mat-making, cobbling, joinery, poultry farming, and net-making. Many of the men indeed learn two trades, so that they will not arrive at a dead end when business is slack.

### The Army of the Blind.

The fourth annual report of the Hostel gives precise figures of the incidence of blindness on our Army during and after the Great War. It should be said here that the definition of blindness recognised by the Ministry of Pensions, in granting an allowance for loss of sight, is "unable to read or write or do ordinary work in an ordinary way." The number of men thus blinded who have come to St. Dunstan's is 1,483, ranked as follows : officers, 75; non-commissioned officers, 249; and privates, 1,159. Of these 690 have completed their re-education as blind men, and already have been established in the business they have entered, and 740 are still making their brave fight against difficulties in the class-rooms and workshops. The remainder, representing under 4 per cent. of those men who have lost their sight in the war, were, for reasons connected with ineradicably bad habits, or seriously impaired mental or physical health, incapable of making good in the world. But there are more to come. It is the hope of Sir Arthur Pearson that these figures will not now receive any considerable addition, but, unfortunately, there is a great number of men who have lost one eye or whose sight has been in some way affected, and for whom training will sooner or later become necessary. It is a tragic fact that it would be folly to overlook the work which possibly lies ahead.

### St. Dunstan's as a Property Owner.

Some mention has been made in a previous article of the large generosity of the public in respect of the work. Large though the sums mentioned are, they will be required to the last penny in the work of the future. Not only money has been given, but also freehold and leasehold houses and estates. The group of properties in and near Regent's Park has been lent, mostly indefinitely, but there are properties at Brighton, Blackheath, St. Leonards-on-Sea, Cheltenham, Ilkley, and elsewhere which have been given outright. These are being usefully employed now, and will be equally usefully employed in the future, those out of London chiefly as recreative and recuperative centres for past and present students at Regent's Park and for those who are physically unfit for training, for St. Dunstan's has done its work thoroughly in the past and intends to do the same in the future. The qualification is to be a blinded sailor or soldier, and the greatest possible good is done with every case, whether trainable or not.

### After-care.

The trained man goes forth from St. Dunstan's; he is thereafter helped and encouraged in every possible way, and it is intended that he shall be helped so long as help is needed.

The after-care system has been planned in a manner which is thorough and businesslike. The country is divided into districts, in each of which there is an inspector, who visits every man at intervals of a few weeks, while experts in the various trades pursued also pay regular visits to the workers. Steps are taken to encourage local demand for goods that have been made, and when local resources are insufficient the articles manufactured are sold at central depôts. Continuity of employment is secured for men whose occupations do not involve the manufacture of articles for sale; raw material is supplied at cost price, and many things are done to assist the blinded professional or working man in his resolute endeavour to make good in the world.

In a great organisation such as this many workers are required for administration. It is, we understand, the intention of the Committee to employ wherever it is possible blind men who have a bent for organisation, in the same way as is being done by the sister institution, the National Institution for the Blind.

### Men That Are More Than Blind.

We have called St. Dunstan's a complex organisation. It is so complex that every side of its work cannot be alluded to in a thick volume, let alone in an article of short limits. We must, however, allude to the Blinded Soldiers' Children Fund (448 of the men were married before they lost their sight and 227 since). A child born to a blinded soldier after his discharge does not come within the Government pension scheme, and of these children 202 receive a weekly allowance up to the age of sixteen through the Children's Fund.

And there is the work of treatment of those blinded men whose arms and hands have been injured; upon the importance of this branch it is not necessary to dwell. Obviously the sense of touch is essential to every blind man; as the annual report says, "his ten fingers become, as it were, ten eyes." There are men who are not only blinded, sometimes having an arm off, sometimes with arms and hands badly injured—a substantial addition to the handicap with which they have to contend. Though sight cannot be restored, it is often possible to repair in some measure other injuries. Forty-four of these special cases have come forward; in seven cases the disabilities other than blindness have been reported upon as quite cured, and in nearly every case encouraging progress has been made, and in spite of the injuries the men are at work—learning to be blind.

**Previous articles appeared on October 18, 25, and November 15, p. 144.**

